# Effectiveness of Vortioxetine Treatment on Depression and Cognitive Functions in Patients with Alzheimer’s Disease: A 12-Month, Retrospective, Observational Study

**DOI:** 10.3390/jpm14090918

**Published:** 2024-08-29

**Authors:** José María García-Alberca, Paz De La Guia, Esther Gris, Silvia Mendoza, María Lopez De La Rica, Miguel Ángel Barbancho, José Pablo Lara, Encarnación Blanco-Reina

**Affiliations:** 1 Alzheimer Research Center and Memory Clinic, Instituto Andaluz de Neurociencia (IANEC), 29012 Málaga, Spain; 2 Brain Health Unit (CIMES), School of Medicine, University of Málaga, IBIMA, 29010 Málaga, Spain; 3 Pharmacology and Therapeutics Department, School of Medicine, University of Málaga, IBIMA, 29010 Málaga, Spain

**Keywords:** Alzheimer’s disease, vortioxetine, cognitive functioning, antidepressants, depressive symptoms, cognitive symptoms

## Abstract

This study aimed to assess the effectiveness of vortioxetine for improving depressive symptoms, cognitive performance, daily and global functioning in patients with Alzheimer’s disease (AD) and major depressive disorder (MDD) in real-world clinical practice. We retrospectively identified 46 AD patients who had received treatment for 12 months with vortioxetine. Drug effects were evaluated at baseline, 4, 8, and 12 months. The primary endpoint was change from baseline in the Hamilton Depression Rating Scale (HDRS) and in the Cornell Scale for Depression in Dementia (CSDD) to month 12. Cognitive and daily and global functioning changes were also evaluated. Significant baseline-to-endpoint improvement in depressive symptom severity was observed (*p* < 0.0001). At month 12, the least-square mean (standard error) change score from baseline was −10.48 (±0.42) on the HDRS and −9.04 (±0.62) on the CSDD. Significant improvements in cognitive performance were observed for the Rey Auditory Verbal Learning Test, the Symbol Digit Modalities Test, the Letter Fluency Test, the Category Fluency Test, and the Trail Making Test-A. Patients also experienced significant improvements in daily and global functioning. Vortioxetine was safe and well tolerated. Patients with AD and MDD receiving vortioxetine showed meaningful improvements in depressive symptoms, cognitive performance, and daily and global functioning over the 12-month treatment period.

## 1. Introduction

Alzheimer’s disease (AD) is the most common cause of dementia globally, accounting for 60% to 80% of cases [1]. In addition to cognitive and functional symptoms, depression is one of the most prevalent neuropsychiatric conditions among patients with AD. Based on accepted diagnostic criteria, the prevalence of depression in patients with AD is estimated to be between 20% and 45% [2]. Depression coexisting with AD has been associated with poor quality of life [3,4], more rapid cognitive decline [5,6], higher use of psychoactive drugs [7], and mortality [8].

In the clinical setting, antidepressants are frequently used for the management of depression in AD [9]. Although some studies have found that antidepressants may have a slight advantage in terms of remission rates [10,11], evidence from systematic reviews and meta-analysis does not provide compelling support for their efficacy [12,13] with no significant differences between antidepressants and placebo in terms of cognitive function [11]. Notwithstanding the considerable reservations pertaining to the potential for adverse effects and efficacy, antidepressants continue to be a frequently prescribed medication in clinical practice [14]. In addition, the treatment of depression in these patients is further complicated by the presence of comorbid medical conditions, the risk of drug interactions, and the fact of their increased vulnerability to side effects of medications [15,16].

Therefore, it would be beneficial to investigate the effects of new antidepressants that have been shown to have a positive impact on cognitive function in order to ascertain their potential efficacy in treating depression in patients with AD. In this sense, vortioxetine is a multimodal antidepressant with a positive effect on cognitive function [17,18] in patients suffering from with a major depressive episode [19,20,21,22,23,24].

Vortioxetine is a selective serotonin reuptake inhibitor and serotonin modulator [25] that has also indirect effects on other systems important for mood and cognitive function, including the noradrenaline, dopamine, histamine, acetylcholine, gamma-aminobutyric acid, and glutamate systems [26,27,28]. It is thought that vortioxetine’s multimodal activity is responsible for its effects on cognitive function [19,22,29], with potential mechanisms including increased glutamate neurotransmission and neuroplasticity in regions such as the prefrontal cortex [19,27].

Vortioxetine has been associated with enhancements in a range of cognitive domains, including global cognition, executive functioning, processing speed, attention, and working memory [22,30]. Furthermore, vortioxetine has also demonstrated efficacy in the treatment of major depressive disorder (MDD) in people with dementia [31,32,33]. An open-label study showed that treatment with vortioxetine led to a notable enhancement in cognitive performance among community-dwelling older adults with mild cognitive impairment and no depressive symptoms [34]. Recent data suggest vortioxetine has unique properties that may be beneficial for elderly patients [35,36]. In addition to improving cognitive function, it has a favorable safety profile in elderly individuals, who are at an increased risk for cardiovascular and cerebrovascular complications [35,36].

The aim of this study was to investigate the effectiveness of vortioxetine in improving depressive symptoms and enhancing cognitive function in patients with MDD and early-stage AD within the context of real-world clinical practice. Overall disease severity, daily functioning, and tolerability were also assessed. To this end, we carried out a retrospective analysis of service provision data over a 12-month period from the Instituto Andaluz de Neurociencia (IANEC) in a sample of people suffering from AD.

## 2. Materials and Methods

### 2.1. Study Design and Participants

This was a retrospective study carried out in patients with a diagnosis of AD who were experiencing a major depressive episode of <6 months’ duration. Patients were attending the Alzheimer Disease Center and Memory Clinic of the Instituto Andaluz de Neurociencia (IANEC), Málaga, Spain, from January 2021 to November 2023.

The diagnosis of AD was made by a qualified professional, specifically a neurologist or psychiatrist, in accordance with the diagnostic criteria established by the National Institute on Aging Alzheimer’s Association workgroups (NIA/AA) [37]. The diagnosis of MDD was made according to *Diagnostic and Statistical Manual of Mental Disorders, Fifth Edition (DSM-5)* criteria [38].

In addition, the patients who participated in the study had no previous history of depression and were experiencing their first major depressive episode treated with antidepressants. In order to assess the possible effectiveness of vortioxetine on cognitive functions in patients with AD and depressive symptoms, we recruited patients in the early-stage of AD, assuming that at this level of impairment the benefits on cognition might become more evident. Patients had a Mini-Mental State Examination (MMSE) [39] total score of 20–24 (i.e., mild dementia) at baseline.

Excluded from the study were patients who had any evidence of focal vascular lesions (such as hematoma), multi-infarct dementia, or history of cerebrovascular disease; primary diagnosis of any psychiatric disorder other than depression; significant neurologic antecedents, such as brain trauma, brain tumors, epilepsy, or inflammatory disease; those with severe medically uncontrolled conditions such as hypothyroidism, cardiovascular, or chronic renal failure; absence of a complete medical history to assess the study variables; presence of a severe sensory disorder (e.g., severe vision and hearing impairment); and those without a legal representative who could sign the informed consent to review their medical records. Patients were taking vortioxetine to treat their depression at their physician’s discretion, in line with the local approved label. This was either as a first-line treatment for the current major depressive episode or as a switch from another antidepressant due to ineffectiveness or side effects.

In accordance with the marketing authorization, the recommended starting dose of vortioxetine was 10 mg/day in patients under 65 years and 5 mg/day for those aged 65 years or above. This was provided as part of the patients′ routine medical care. The dosage of vortioxetine could be adjusted within the approved dose range of 5–20 mg/day at the discretion of the treating physician, based on the patient′s response to the treatment. Use of other antidepressants was not permitted during the study. Patients receiving treatment for dementia, including acetylcholinesterase inhibitors and memantine, had to have been on a stable dose for ≥3 months before screening. No patients changed their antidementia drug or combined treatment with AChEI plus memantine during the study. Patients also maintained a stable dose of other medications such as antihypertensives, diuretics, lipid-reducing agents, and antidiabetic drugs. During the study, some patients were also administered occasional doses of anxiolytics or hypnotics for the symptomatic treatment of anxiety or sleep problems. No patients required treatment with antipsychotics throughout the course of the study.

The study was carried out using a sample of patients selected from the IANEC records database. As part of the routine clinical follow-up, patients at the IANEC undergo an initial evaluation upon admission and subsequently evaluated on a regular basis. The data were collected by clinical researchers from the patient’s medical records.

The study was conducted in accordance with the Declaration of Helsinki and Good Clinical Practice guidelines and was approved by the Málaga Clinical Research Ethics Committee (protocol code 11/2023 PI13, approved on 4 December 2023). Informed consent was obtained from patients or their representative caregivers prior to their inclusion.

### 2.2. Measures

Assessments were conducted at four time points: at baseline and at months 4 (±1), 8 (±1), and 12 (±1) of vortioxetine treatment.

Severity of depressive symptoms was assessed using the Hamilton Depression Rating Scale (HDRS) [40] and the Cornell Scale for Depression on Dementia (CSDD) [41]. The HDRS (17-items version) was completed at the end of a semi-structured interview by a trained clinician and it focuses basically on the somatic and behavioral components of depression. A higher score indicates a higher level of depression. The CSDD is a 19-item interview. Scores above 10 indicate probable major depressive disorder, and scores above 18 indicate definite major depression.

Cognitive performance was assessed using the Rey Auditory Verbal Learning Test (RAVLT) [42], the Symbol Digit Modalities Test (SDMT) [43], the Trail Making Test (forms A and B) [44], and the Letter and Category Fluency Tests (LFT, CFT) [45].

The RAVLT is a cognitive assessment tool designed to evaluate verbal episodic memory. It uses two different lists of 15 words (List A and List B) to test immediate and delayed recall. Five presentations of list A are given, each followed by attempted recall. Immediate recall (IR) is assessed by asking the patient to recall as many words as possible from List A (Trials I to V). In this study, the sum total of words recalled in Trials I to V was used to measure the total encoding of IR.

The SDMT is a well-established tool for assessing processing speed and sustained attention. The subject is shown a page with a key that matches the single digits 1–9 with nine symbols. Rows below contain only symbols. The subject is required to write or orally report the correct number in the spaces provided. Following the completion of the initial 10 items with guidance, the subject is timed to determine the number of responses that can be made within a 90 s period.

The Trail Making Test (TMT) is used to evaluate an individual′s ability to flexibly switch attention between competing task-set representations. The TMT is composed of two parts, TMT-A and TMT-B. The TMT-A asks the participant to draw lines and connect circled numbers in a numerical sequence. In the TMT-B, the participant is instructed to connect circled numbers and letters in an alternating sequence of numerical and alphabetical order. The participant is required to complete both task components as efficiently and exactly as possible.

The CFT and LFT were used to assess verbal fluency. In the CFT, participants were requested to provide as many animals as possible within a one-minute timeframe. In the LFT, participants were instructed to say as many words as possible that begin with the letter P for a period of one minute.

The assessment of daily functioning was conducted using the Interview for Deterioration in Daily Living (IDDL) [46]. This scale is composed of 33 items that assess both self-care (items 1–16) and complex instrumental activities (items 17–33). In this study, we obtained a total IDDD score (ranging from 33 to 99 points, where higher scores indicate worse functional ability), a basic activities score (IDDD-B, ranging from 16 to 48), and an instrumental activities score (IDDD-I, ranging from 17 to 51).

The overall disease severity and clinical improvement were assessed using the Clinical Global Impression—Severity of illness scale (CGI-S) and the Clinical Global Impression—Improvement scale (CGI-I) [47,48]. The CGI-S assesses the severity of the disease at the present time. It uses a single item on a 7-value Likert scale from 1 (normal, not at all ill) to 7 (among the most extremely ill patients). The CGI-I assesses improvement due to therapeutic interventions. It ranges from 1 (very much improved) to 7 (very much worse).

Data on treatment-emergent adverse events (TEAEs) were collected and recorded in the medical records by their treating physicians at the time of their occurrence.

### 2.3. Efficacy Outcomes

There were two primary efficacy variables: the change from baseline to month 12 in the HDRS and the change from baseline to month 12 in the CSDD. The global assessment of change in RAVLT, SDMT, TMT-A, TMT-B, CFT, LFT, IDDD, CGI-S and CGI-I were secondary clinical efficacy variables.

### 2.4. Statistical Analysis

At baseline, demographic and clinical data are reported using mean and standard deviation for continuous variables, and number and percentage for categorical or binary variables. The primary efficacy endpoints were analyzed over time as change from baseline to month 12 using a mixed model for repeated measurements (MMRM) analysis including month as fixed factor and baseline HDRS and CSDD total scores as covariates. Exploratory analyses of change in HDRS and CSDD total scores were also performed: (i) in the subgroup of patients with severe cognitive impairment at baseline (i.e., those with a baseline SDMT score at least 0.5 standard deviations [SD] below the mean score at baseline); and (ii) using MMSE score at screening instead of HDRS and CSDD total scores as a baseline covariate.

We used a similar MMRM model to analyze the secondary endpoints. Standardized Effect Size (SES) was also calculated for the change in clinical variables scores from baseline. The differences from baseline to month 12 were estimated using least-squares (LS) mean change (with 95% confidence interval [CI] or standard error [SE] of the mean).

Rates of response and remission were calculated, with response defined as a ≥50% reduction in HDRS total score from baseline and remission defined as an HDRS total score ≤ 7. Furthermore, the rates of response and remission were evaluated through the use of Clinical Global Impressions (CGI) scores.

Response was defined as a CGI-I score of ≤2 and remission as a CGI-S of ≤2 [33]. We used descriptive statistics to summarize the safety endpoints.

Statistical analyses were conducted using the Statistical Package for the Social Sciences Software (SPSS 28.0, IBM Corporation, Armonk, New York, NY, USA). Significance was set at *p* < 0.05.

## 3. Results

### 3.1. Baseline Characteristics of Participants

The patient sociodemographics and clinical characteristics at baseline are presented in Table 1. A total of 46 patients met the inclusion criteria and their data were available for analysis (32 female, 14 male). Patients had a mean age of 75.1 years (range 65–95) and mean years of education of 6.6 (range 4–17). All patients were Caucasian. 

Approximately two-thirds of patients (69.6%) received vortioxetine as first-line treatment for the current depressive episode. Of the 14 patients switching to vortioxetine from another antidepressant, most (12, 85.7%) were switching due to the lack of effectiveness of their prior therapy, and 2 due to the lack of tolerability. In these patients, the most commonly prescribed antidepressants were an SSRI or SNRI (*n* = 13). Escitalopram was the most commonly previous antidepressant medication (*n* = 7) followed by sertraline (*n* = 4), and trazodone (*n* = 4). At some point during the course of the study, 19 (41.3%) patients were administered concomitant treatment with benzodiazepines, while 9 (19.6%) patients were treated with hypnotics.

Mean (SD) duration of the current major depressive episode was 21.3 (±3.36) weeks.

A total of 42 patients (91%) exhibited comorbid medical conditions at the baseline assessment. All subjects were receiving antidementia drugs, most commonly rivastigmine patches (25 patients, 54.3%), which were administered as monotherapy in most cases (*n* = 21, 43.7%).

Baseline assessment scores indicated that the study population had moderate to severe depression (mean [SD] HDRS total score, 17.83 [±4.21] and mean [SD] CSDD total score, 18.35 [±4.52]) and early-stage AD (mean [SD] MMSE score, 21.24 [±1.06]). Baseline CGI-S scores were indicative of moderate to severe illness.

### 3.2. Vortioxetine Dosing 

**Figure 1:**
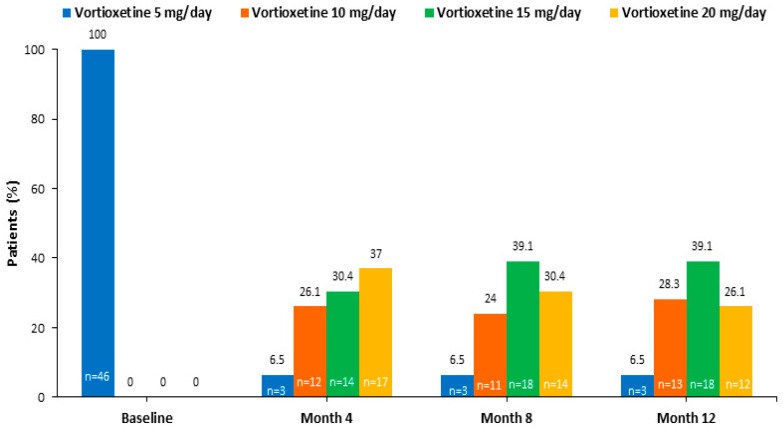
Vortioxetine dosage throughout the study.

The mean (SD) dose of vortioxetine over the 12-month study period was 14.2 (±3.52) mg/day. All patients received vortioxetine 5 mg/day during the first two weeks of treatment. From weeks 2 to 6, all patients received vortioxetine 10 mg/day. From week 6 to the end of the study, the vortioxetine dose was individualized for each patient according to the therapeutic response and could be increased up to 20 mg. At month 12, 12 patients (26.1%) were taking the maximum recommended dose of 20 mg/day (Figure 1).

### 3.3. Depressive Symptoms

**Figure 2:**
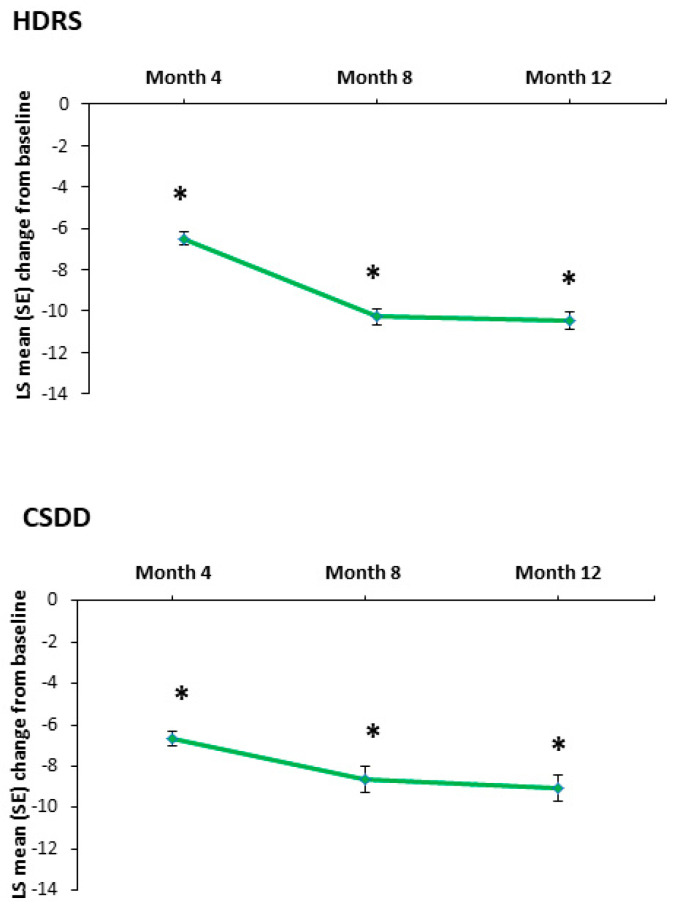
LS mean (SE) change from baseline in the Hamilton Depression Rating Scale (HDRS) and the Cornell Scale for Depression on Dementia (CSDD) total scores. * *p* < 0.0001 vs baseline. LS = least-squares; SE = standard error.

A statistically significant improvement in the severity of depressive symptoms, as measured by the HDRS and the CSDD total scores, was seen at all assessment time points (*p* < 0.0001) (Table 2). The LS mean (SE) change from baseline to month 12 was −10.48 (± 0.42) on the HDRS and −9.04 (±0.62) on the CSDD (Figure 2). At month 12, the SES for change from baseline was 0.89 on the HDRS total score and 0.78 on the CSDD total score.

In the predefined subgroup of patients with more severe cognitive impairment at baseline (i.e., baseline SDMT score > 0.5 SDs below the mean baseline score; *n*= 13), the LS mean (SE) change from baseline to month 12 was −10.31 (± 0.78) on the HDRS and −11.31 (±0.96) on the CSDD. This result is similar to the change observed in the overall study population, indicating that the improvement in depressive symptoms was not adversely affected in patients with more severe cognitive impairment at baseline.

Using the MMSE score as a baseline covariate, the LS mean (SE) change from baseline to month 12 was −10.48 (±0.51) on the HDRS and −9.04 (±0.63) on the CSDD, indicating the same significant improvement in severity of depressive symptoms when adjusting for baseline dementia severity.

**Figure 3:**
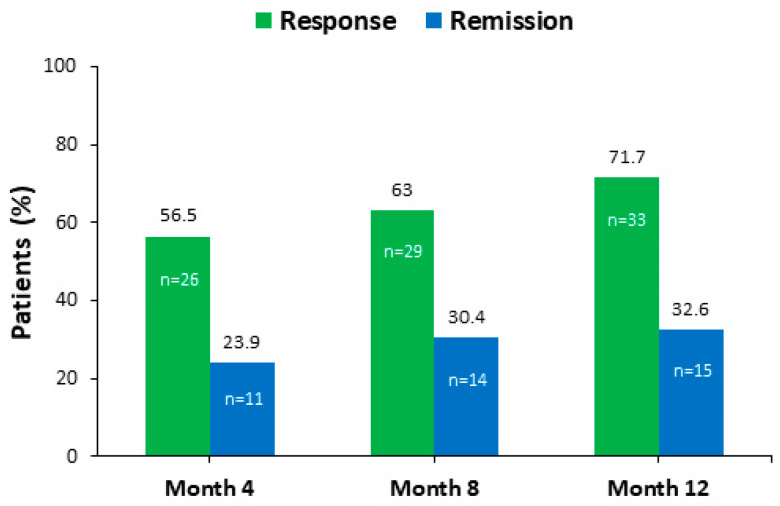
Response and remission rates on the Hamilton Depression Rating Scale (HDRS) score. Response was defined as a 50% reduction of HDRS total score from baseline. Remission was defined as an HDRS total score ≤ 7 points.

At month 12, 33 (71.7%) patients achieved a response to treatment (≥50% reduction in the HDRS total score from baseline) and 15 (32.6%) were in remission from their depressive symptoms (HDRS total score ≤ 7) (Figure 3).

### 3.4. Cognitive Function

**Figure 4:**
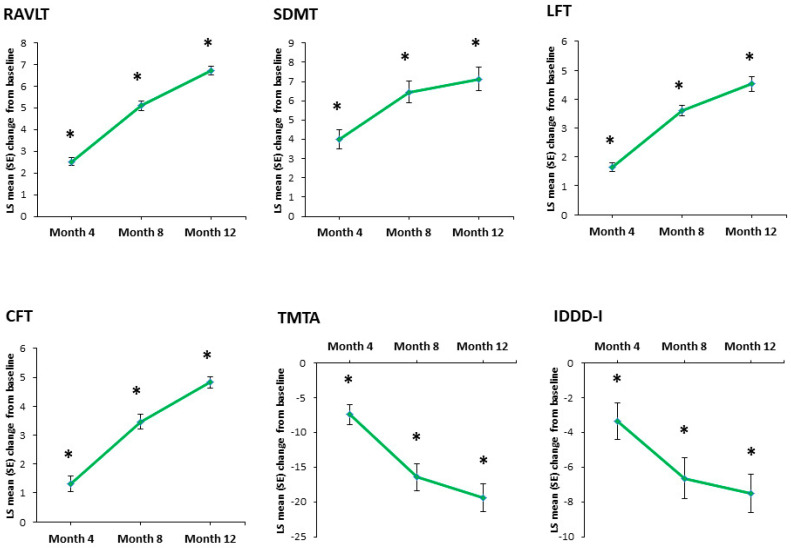
Significant LS mean (SE) change from baseline in cognitive and functional test scores. * *p <* 0.0001 vs. baseline. LS = least squares; SE *=* standard error; RAVLT = Rey Auditory Verbal Learning Test; SDMT = Symbol Digit Modalities Test; LFT = Letter Fluency Test; CFT = Category Fluency Test; TMT-A = Trail Making Test part A; IDDD-I = Interview for Deterioration in Daily Living Activities in Dementia, instrumental activities.

Regarding cognitive function, vortioxetine treatment yielded statistically significant improvement in most cognitive measures at all assessment time points (*p <* 0.0001) (Table 2, Figure 4). More precisely, the LS mean (SE) change from baseline to month 12 was 6.72 (±0.16) on the RAVLT, 7.13 (±0.62) on the SDMT, 4.83 (±0.19) on the CFT, 4.52 (±0.26) on the LFT, and −19.39 (±1.98) on the TMT-A (*p* < 0.0001 in all cases). At month 12, the SES for change from baseline ranged from medium to large (Table 2).

### 3.5. Daily Functioning

The MMRM did not show a statistically significant improvement in daily functioning as measured with the IDDD total score at month 12 (*p* = 0.715). However, a significant improvement was observed in instrumental activities of daily living as measured by the IDDD-I total score (LS mean [SE] change from baseline to month 12 was −7.5 [±2.32]) (Table 2, Figure 4).

### 3.6. Overall Disease Severity and Impact on Global Functioning

**Figure 5:**
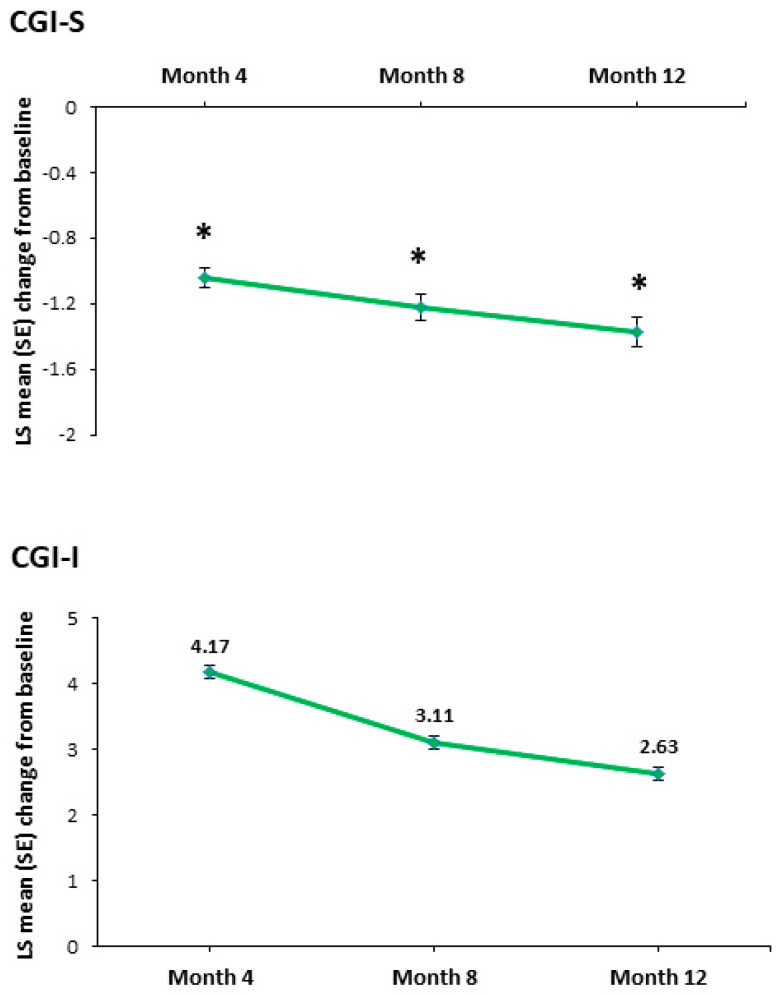
LS mean (SE) change in Clinical Global Impression—Severity (CGI-S) score from baseline (* *p <* 0.0001) and LS mean (SE) Clinical Global Impression—Improvement (CGI-I) score over time.

A significant change in the CGI-S total score from baseline was seen at all time points (*p <* 0.0001) (Table 2). The LS mean (SE) change from baseline to month 12 was −1.37 (±0.09) (Figure 5). Overall improvement in patients’ condition was also observed during the 12 months of vortioxetine treatment. The LS mean (SE) CGI-I score at month 12 was 2.63 (±0.09) (Figure 5).

Symptomatic and functional response (i.e., a CGI-I score ≤ 2) was achieved by 15 patients (32.6%) at month 4, 17 patients (37%) at month 8, and 21 patients (45.7%) at month 12. Remission (i.e., a CGI-S score ≤ 2) was observed in 4 patients (8.7%) at month 4, 6 patients (13%) at month 8, and 8 patients (17.4%) at month 12.

### 3.7. Safety Analysis

Vortioxetine was well tolerated and no unexpected TEAEs were reported (Table 3). During the 12-month treatment period, 27 TEAEs were reported by 17 patients (37%). The most commonly reported TEAEs during treatment were nausea (10.9%) and headache (8.7%). Most TEAEs were transient, were of mild-to-moderate intensity, and resolved spontaneously. No deaths or serious TEAEs occurred during the study.

## 4. Discussion

This retrospective study assessed the effects of vortioxetine on depression and on cognitive function in patients with early AD and MDD. Vortioxetine demonstrated clinical efficacy in improving depressive symptoms, cognitive performance, and daily functioning in actual clinical practice.

Vortioxetine was well tolerated and adverse events were transient and predominantly of mild to moderate intensity in line with the established favorable tolerability profile of vortioxetine in patients with major depressive disorder [49]. The majority of patients (69.6%) were undergoing treatment with vortioxetine as a primary intervention for their current depressive episode.

Following the initiation of treatment at the recommended dose for elderly patients of 5 mg/day during the first two weeks, the vortioxetine dose was adjusted individually for each patient according to the therapeutic response. Upon completion of the study, two-thirds of the patients were receiving vortioxetine at doses of 20 mg per day and 15 mg per day, with no adverse effects observed in this elderly patient population. A significant and sustained improvement in depressive symptoms was observed after 12 months of treatment.

Over two-thirds of patients demonstrated a positive response to treatment, with one-third achieving remission. Regardless of the severity of cognitive impairment at baseline, improvements in depressive symptoms were observed. The results also indicate that, at the end of the study, vortioxetine had a beneficial effect on cognitive functioning, as evidenced by significant and clinically meaningful improvements in the RAVLT, SDMT, CFT, LFT, and TMT-A total scores from baseline at all time points. Treatment appears to benefit performance on a variety of cognitive domains: working memory, verbal episodic memory, spatial perception, visual scanning, executive functions, processing speed, and selective and sustained attention over the 12 months of vortioxetine treatment. In this study, we recruited patients with a clinical diagnosis of early stage of dementia (MMSE score of 20–24 at baseline) on the assumption that at this level of impairment, the procognitive benefits of an antidepressant on cognition might be more evident [29,50]. 

In addition to improvements in depressive symptom severity and cognitive function, a significant improvement in patients’ daily functioning was observed in instrumental activities of daily living as measured by the IDDD-I total score after 12 months of vortioxetine treatment. The benefits observed in objective measurements were further supported by clinician-based assessments of improvements in disease state and severity. In this regard, most patients exhibited overall clinical improvement evidenced by the CGI-I and CGI-S total scores during the 12 months of study period. 

Our results are consistent with those reported in other previous studies of vortioxetine in patients with MDD suffering from AD. In a 12-month, open-label, observational study of vortioxetine in patients with mild AD and depressive symptoms [26], clinical improvements were observed. Patients who received vortioxetine exhibited significantly greater improvements in both mood symptoms and in cognitive function compared to those who received other antidepressants. Similarly, in a 6-month open-label study [29], community-dwelling older adults with mild cognitive impairment treated with vortioxetine showed significant and sustained improvements in cognitive function. In a large postmarketing surveillance study, a subgroup analysis was undertaken to evaluate the effectiveness and tolerability of vortioxetine in patients with MDD and comorbid AD [27]. Results showed that sustained and clinically meaningful improvements in depressive and cognitive symptoms were observed during the 6 months of vortioxetine treatment. 

More recently, the effectiveness of vortioxetine in improving depressive symptoms and cognitive performance in patients with MDD and early-stage dementia was investigated in a 12-week open-label study [28]. Significant improvements in depressive symptom severity and in cognitive performance were observed.

In comparison to previous findings, in the present study, cognitive performance was assessed using a multifactorial neuropsychological evaluation that broadly covered aspects of cognitive function. Furthermore, our continued evaluation enabled us to closely monitor clinical changes over an extended follow-up period of 12 months. It is noteworthy that the objective cognitive performance was maintained over the 12-month period of vortioxetine treatment, suggesting that the clinical benefit of vortioxetine may be enhanced with prolonged use.

Unlike previous studies with vortioxetine in patients with dementia, we considered it interesting to ascertain whether the statistical significance observed in the cognitive tests corresponded to clinically meaningful differences in the cognitive domains assessed. In this sense, standardized effect sizes could be used to contextualize the magnitude of the observed effect on cognitive dysfunction. In accordance with this, the clinical significance of the effect of vortioxetine on neuropsychological test scores could be supported by the magnitude of the effect sizes obtained with a median of 0.75. The objective neuropsychological tests demonstrated cognitive improvements, which were further validated by physician-based assessments of improvements in disease state. The CGI-I overall assessment demonstrated that approximately 50 patients experienced global improvements in disease state at month 12.

The clinical benefits observed during treatment with vortioxetine in these patients could be explained by its multimodal mechanism of action [34]. It is thought that vortioxetine provides direct agonism at the 5-HT1A receptor, partial agonism at the 5-HT1B receptor, and antagonism at the 5-HT3, 5-HT1D, and 5-HT7 receptors [34,51].

The aforementioned effects on serotonergic receptors may serve to enhance the antidepressant effects resulting from serotonin transporter protein inhibition. Furthermore, it is postulated that some of these serotonergic receptor interactions may facilitate the release of other neurotransmitters, including norepinephrine, dopamine, acetylcholine, histamine, gamma-aminobutyric acid, and glutamate [34,35]. This distinctive pharmacodynamic profile is believed to be responsible for the antidepressant and procognitive effects of vortioxetine observed in rat models [51,52,53].

Treatment with vortioxetine for up to 12 months was associated with the known favorable safety profile [49]. The observed proportion of subjects with adverse events in our study (37%) was lower than that estimated in a pooled safety analysis of 11 RCTs in MDD patients (64.9% of patients with treatment-emergent adverse events; average vortioxetine dose 5 mg/day, duration 6–8 weeks) [49]. Adverse events were mostly transient and mild to moderate in intensity, being nausea (10.9%) and headache (8.7%) the most common, consistent with vortioxetine’s expected side effect profile from clinical studies [49].

A strength of this study is that it was conducted in a cohort of patients with AD and MDD receiving treatment in routine clinical practice with a long follow-up period. There is an increasing need to disseminate data on the actual effectiveness of treatments and to confirm or disconfirm results derived from clinical efficacy trials. Furthermore, our data are derived from a distinct health and geographical context, which differentiates it from other published studies. A further strength is that patients underwent a comprehensive evaluation with widely used scales focused on reducing observation bias. Some limitations should be noted. Firstly, this was an observational and retrospective study. It is therefore possible that the results may be affected by this circumstance, and that as a result we are unable to conclude on causality. Secondly, this is a study of a single center, which resulted in a limited number of subjects being enrolled and a small sample size. Therefore, the results should be considered indicative. However, we believe that the data have proven to be clear and sufficient to reach statistical significance. A further limitation of the study was that all subjects were Caucasian. Consequently, it cannot be assumed that the safety and efficacy of treatments among this small subgroup generalize to other groups [54].

## 5. Conclusions

To conclude, findings of this study showed the real-world effectiveness and tolerability of vortioxetine in patients with MDD and early-stage AD. Clinically meaningful improvements in depressive symptoms, cognitive symptoms, and global functioning were observed over the 12 months of vortioxetine treatment. These results may have clinical significance in choosing drugs for depressed patients with AD and should inspire the design of future long-term randomized controlled studies that contribute to supporting the use of vortioxetine for improving cognitive function in AD patients. In addition, this study could aid clinicians in the treatment of AD, benefit the patients concerned, and provide reliable references for broad application.

## Figures and Tables

**Table 1:** Demographic and clinical characteristics at baseline.

Variable	Vortioxetine (*n* = 46)
Demographic characteristics	
Age, years	75.1 ± 5.37
Female	32 (69.6)
Education, years	6.6 ± 2.1
Married	18 (39.1)
Single/divorced	4 (8.7)
Widowed	24 (52.2)
Comorbid medical conditions	
Hypertension	17 (36.9)
Diabetes mellitus	9 (19.6)
Type 2 diabetes mellitus	5 (10.9)
Dyslipidemia	15 (32.6)
Heart disease	7 (15.2)
Duration of current MDE, weeks	21.3 ± 3.36
Prior antidepressant treatment	14 (30.4)
SSRI	8 (57.1)
SNRI	5 (35.7)
Other	4 (28.6)
Clinical assessment scores	
HDRS	17.83 ± 4.21
CSDD	18.35 ± 4.52
MMSE	21.24 ± 1.06
RAVLT	24.65 ± 1.43
SDMT	22.33 ± 3.45
CFT	8.89 ± 0.95
LFT	10.52 ± 2.02
TMT-A	134.98 ± 20.85
TMT-B	229.78 ± 33.80
IDDD (total)	50.20 ± 13.16
IDDD-B	19.89 ± 3.14
IDDD-I	33.28 ± 12.40
CGI-S	4.30 ± 0.69

Data are presented as means ± SD or number (%). MDE = major depressive episode; SSRI = selective serotonin reuptake inhibitor; SNRI = selective serotonin–noradrenaline reuptake inhibitor; HDRS = Hamilton Depression Rating Scale; CSDD = Cornell Scale for Depression in Dementia; MMSE = Mini-Mental State Examination; RAVLT = Rey Auditory Verbal Learning Test; SDMT = Symbol Digit Modalities test; CFT = Category Fluency Test; LFT = Letter Fluency Test; TMT-A = Trail Making Test part A; TMT-B = Trail Making Test part B; IDDD = Interview for Deterioration in Daily Living Activities in Dementia; IDDD-B = Interview for Deterioration in Daily Living Activities in Dementia, basic activities; IDDD-I = Interview for Deterioration in Daily Living Activities in Dementia, instrumental activities; CGI-S = Clinical Global Impression—Severity Scale.

**Table 2:** LS mean (SE) change from baseline in clinical variable total scores after 4, 8, and 12 months of vortioxetine treatment.

Variable	Month 4	Month 8	Month 12	SES at Month 12
HDRS	−6.50 (0.28) *	−10.28 (0.37) *	−10.48 (0.42) *	0.89
CSDD	−6.65 (0.36) *	−8.63 (0.63) *	−9.04 (0.62) *	0.78
RAVLT	2.52 (0.15) *	5.11 (0.20) *	6.72 (0.16) *	0.93
SDMT	4.00 (0.50) *	6.46 (0.57) *	7.13 (0.62) *	0.66
CFT	1.30 (0.27) *	3.46 (0.25) *	4.83 (0.19) *	0.75
LFT	1.65 (0.14) *	3.61 (0.15) *	4.52 (0.26) *	0.82
TMT-A	−7.41 (1.40) *	−16.41 (1.92) *	−19.39 (1.98) *	0.59
TMT-B	−2.74 (1.28)	−3.54 (1.74)	−4.35 (2.10)	0.05
IDDD	−0.85 (1.71)	−2.46 (1.84)	−2.22 (1.82)	0.01
IDDD-B	2.68 (1.21)	2.76 (1.34)	0.37 (1.35)	0.02
IDDD-I	−3.35 (1.04)	−6.63 (1.39) **	−7.5 (1.32) **	0.75
CGI-S	−1.04 (0.06) *	−1.22 (0.08) *	−1.37 (0.09) *	0.74

* *p* < 0.0001, ** *p* < 0.05. LS = least-squares; SE = standard error; SES =standardized effect size; HDRS = Hamilton Depression Rating Scale; CSDD = Cornell Scale for Depression in Dementia; RAVLT = Rey Auditory Verbal Learning Test; SDMT = Symbol Digit Modalities Test; CFT = Category Fluency Test; LFT = Letter Fluency Test; TMT-A = Trail Making Test part A; TMT-B = Trail Making Test part B; IDDD = Interview for Deterioration in Daily Living Activities in Dementia; IDDD-B = Interview for Deterioration in Daily Living Activities in Dementia, basic activities; IDDD-I = Interview for Deterioration in Daily Living Activities in Dementia, instrumental activities; CGI-S = Clinical Global Impression—Severity Scale.

**Table 3:** Summary of TEAEs reported.

	Nº of Patients (%)	Nº of Events
Any AE	17 (36.9)	27
Any AEs occurring in ≥2 patients Nausea	5 (10.9)	5
Headache	4 (8.7)	4
Dizziness	3 (6.5)	3
Constipation Diarrhea	3 (6.5) 3 (6.5)	3 3
Insomnia	2 (4.3)	2
Somnolence	2 (4.3)	2
Vertigo	2 (4.3)	2
Agitation	2 (4.3)	2
Decreased appetite	2 (4.3)	2

TEAE = treatment-emergent adverse event.

## Data Availability

The datasets used and/or analyzed during the current study are available from the corresponding author upon reasonable request.

## References

[1] (2022). 2022 Alzheimer’s disease facts and figures. Alzheimer’s Dement..

[2] Leung D.K.Y., Chan W.C., Spector A., Wong G.H.Y. (2021). Prevalence of depression, anxiety, and apathy symptoms across dementia stages: A systematic review and meta-analysis. Int. J. Geriatr. Psychiatry.

[3] Tatsumi H., Nakaaki S., Torii K., Shinagawa Y., Watanabe N., Murata Y., Sato J., Mimura M., Furukawa T.A. (2009). Neuropsychiatric symptoms predict change in quality of life of Alzheimer disease patients: A two-year follow-up study. Psychiatry Clin. Neurosci..

[4] Burke A.D., Goldfarb D., Bollam P., Khokher S., Khokher S. (2019). Diagnosing and treating depression in patients with Alzheimer’s disease. Neurol. Ther..

[5] Arnaud A.M., Brister T.S., Duckworth K., Foxworth P., Fulwider T., Suthoff E.D., Werneburg B., Aleksanderek I., Reinhart M.L. (2022). Impact of major depressive disorder on comorbidities: A systematic literature review. J. Clin. Psychiatry.

[6] Holtzer R., Scarmeas N., Wegesin D.J., Albert M., Brandt J., Dubois B., Hadjigeorgiou G.M., Stern Y. (2005). Depressive symptoms in Alzheimer’s disease: Natural course and temporal relation to function and cognitive status. J. Am. Geriatr. Soc..

[7] Dafsari F.S., Jessen F. (2020). Depression–An underrecognized target for prevention of dementia in Alzheimer’s disease. Transl. Psychiatry.

[8] Mehta K.M., Yaffe K., Langa K.M., Sands L., Whooley M.A., Covinsky K.E. (2003). Additive effects of cognitive function and depressive symptoms on mortality in elderly community living adults. J. Gerontol. A Biol. Sci. Med. Sci..

[9] Rabins P.V., Rovner B.W., Rummans T., Schneider L.S., Tariot P.N. (2017). Guideline watch (October 2014): Practice guideline for the treatment of patients with Alzheimer’s disease and other dementias. Focus.

[10] Su J.A., Chang C.C., Wang H.M., Chen K.J., Yang Y.H., Lin C.Y. (2019). Antidepressant treatment and mortality risk in patients with dementia and depression: A nationwide population cohort study in Taiwan. Ther. Adv. Chronic. Dis..

[11] He Y., Li H., Huang J., Huang S., Bai Y., Li Y., Huang W. (2021). Efficacy of antidepressant drugs in the treatment of depression in Alzheimer disease patients: A systematic review and network meta-analysis. J. Psychopharmacol..

[12] Orgeta V., Tabet N., Nilforooshan R., Howard R. (2017). Efficacy of antidepressants for depression in Alzheimer’s disease: Systematic review and meta-Analysis. J. Alzheimer’s Dis..

[13] Dudas R., Malouf R., McCleery J., Dening T. (2018). Antidepressants for treating depression in dementia. Cochrane Database Syst. Rev..

[14] Livingston G., Sommerlad A., Orgeta V., Costafreda S.G., Huntley J., Ames D., Ballard C., Banerjee S., Burns A., Cohen-Mansfield J. (2017). Dementia prevention, intervention, and care. Lancet.

[15] Lyketsos C.G., Lopez O., Jones B., Fitzpatrick A.L., Breitner J., DeKosky S. (2002). Prevalence of neuropsychiatric symptoms in dementia and mild cognitive impairment: Results from the cardiovascular health study. J. Am. Med. Assoc..

[16] Goodarzi Z., Mele B., Guo S., Hanson H., Jette N., Patten S., Pringsheim T., Holroyd- Leduc J. (2016). Guidelines for dementia or Parkinson’s disease with depression or anxiety: A systematic review. BMC Neurol..

[17] Blumberg M.J., Vaccarino S.R., McInerney S.J. (2019). Procognitive effects of antidepressants and other therapeutic agents in major depressive disorder: A systematic review. J. Clin. Psychiatry.

[18] Salagre E., Solé B., Tomioka Y., Fernandes B.S., Hidalgo-Mazzei D., Garriga M., Jimenez E., Sanchez-Moreno J., Vieta E., Grande I. (2017). Treatment of neurocognitive symptoms in unipolar depression: A systematic review and future perspectives. J. Affect. Disord..

[19] Baune B.T., Sluth L.B., Olsen C.K. (2018). The effects of vortioxetine on cognitive performance in working patients with major depressive disorder: A short-term, randomized, double-blind, exploratory study. J. Affect. Disord..

[20] Huang I.C., Chang T.S., Chen C., Sung J.Y. (2022). Effect of vortioxetine on cognitive impairment in patients with major depressive disorder: A systematic review and metaanalysis of randomized controlled trials. Int. J. Neuropsychopharmacol..

[21] Mahableshwarkar A.R., Zajecka J., Jacobson W., Chen Y., Keefe R.S. (2015). A randomized, placebo-controlled, active-reference, double-blind, flexible-dose study of the efficacy of vortioxetine on cognitive function in major depressive disorder. Neuropsychopharmacology.

[22] McIntyre R.S., Lophaven S., Olsen C.K. (2014). A randomized, double-blind, placebo-controlled study of vortioxetine on cognitive function in depressed adults. Int. J. Neuropsychopharmacol..

[23] McIntyre R.S., Harrison J., Loft H., Jacobson W., Olsen C.K. (2016). The effects of vortioxetine on cognitive function in patients with major depressive disorder: A meta-analysis of three randomized controlled trials. Int. J. Neuropsychopharmacol..

[24] McIntyre R.S., Florea I., Tonnoir B., Loft H., Lam R.W., Christensen M.C. (2017). Efficacy of vortioxetine on cognitive functioning in working patients with major depressive disorder. J. Clin. Psychiatry.

[25] Bang-Andersen B., Ruhland T., Jørgensen M., Smith G., Frederiksen K., Jensen K.G., Zhong H., Nielsen S.M., Hogg S., Mørk A. (2011). Discovery of 1-[2-(2,4-dimethylphenylsulfanyl)phenyl] piperazine (Lu AA21004): A novel multimodal compound for the treatment of major depressive disorder. J. Med. Chem..

[26] Pehrson A.L., Sanchez C. (2014). Serotonergic modulation of glutamate neurotransmission as a strategy for treating depression and cognitive dysfunction. CNS Spectr..

[27] Sanchez C., Asin K.E., Artigas F. (2015). Vortioxetine, a novel antidepressant with multimodal activity: Review of preclinical and clinical data. Pharmacol. Ther..

[28] Stahl S.M. (2015). Modes and nodes explain the mechanism of action of vortioxetine, a multimodal agent (MMA): Enhancing serotonin release by combining serotonin (5HT) transporter inhibition with actions at 5HT receptors (5HT1A, 5HT1B, 5HT1D, 5HT7 receptors). CNS Spectr..

[29] Bennabi D., Haffen E., Van Waes V. (2019). Vortioxetine for cognitive enhancement in major depression: From animal models to clinical research. Front. Psychiatry.

[30] Mattingly G.W., Ren H., Christensen M.C., Katzman M.A., Polosan M., Simonsen K., Hammer-Helmich L. (2022). Effectiveness of Vortioxetine in Patients with Major Depressive Disorder in Real-World Clinical Practice: Results of the RELIEVE Study. Front. Psychiatry.

[31] Cumbo E., Cumbo S., Torregrossa S., Migliore D. (2019). Treatment effects of vortioxetine on cognitive functions in mild Alzheimer’s disease patients with depressive symptoms: A 12 month, open-label, observational study. J. Prev. Alzheimer’s Dis..

[32] Cumbo E., Adair M., Åstrom D.O., Christensen C. (2023). Effectiveness of vortioxetine in patients with major depressive disorder and comorbid Alzheimer’s disease in routine clinical practice: An analysis of a post-marketing surveillance study in South Korea. Front. Aging Neurosci..

[33] Christensen M.C., Schmidt S.N., Grande I. (2023). Effectiveness of vortioxetine in patients with major depressive disorder and early-stage dementia: The MEMORY study. J. Affect. Disord..

[34] Tan S.N., Tan C. (2021). Vortioxetine improves cognition in mild cognitive impairment. Int. Clin. Psychopharmacol..

[35] Danielak D. (2021). Vortioxetine in management of major depressive disorder–A favorable alternative for elderly patients?. Expert. Opin. Pharmacother..

[36] Bishop M.M., Fixen D.R., Linnebur S.A., Pearson S.M. (2021). Cognitive effects of vortioxetine in older adults: A systematic review. Ther. Adv. Psychopharmacol..

[37] McKhann G.M., Knopman D.S., Chertkow H., Hyman B.T., Jack C.R., Kawas C.H., Klunk W.E., Koroshetz W.J., Manly J.J., Mayeux R. (2011). The diagnosis of dementia due to Alzheimer’s disease: Recommendations from the National Institute on Aging Alzheimer’s association workgroups on diagnostic guidelines for Alzheimer’s disease. Alzheimer’s Dement..

[38] American Psychiatric Association (2013). Diagnostic and Statistical Manual of Mental Disorders (DSM-5^®^).

[39] Folstein M.F., Folstein S.E., McHugh P.R. (1975). Mini-mental state. A practical method for grading the cognitive state of patients for the clinician. J. Psychiatr. Res..

[40] Hamilton M.J. (1960). A rating scale for depression. Neurol. Neurosurg. Psychiatry.

[41] Yesavage J.A., Brink T.L., Rose T.L., Lum O., Huang V., Adey M., Leirer V.O. (1982). Development and validation of a geriatric depression screening scale: A preliminary report. J. Psychiatr. Res..

[42] Rey A. (1958). L’Examen Clinique in Psychologie.

[43] Smith A. (1982). Symbol Digit Modalities Test: Manual.

[44] Reitan R.M., Wolfson D. (1983). The Halstead-Reitan Neuropsychological Test Battery: Theory and Clinical Interpretation.

[45] Peña-Casanova J. (2005). Test Barcelona Revisado.

[46] Teunisse S., Derix M.M. (1991). Measurement of activities of daily living in patients with dementia living at home: Development of a questionnaire. Tijdschr. Gerontol. Geriatr..

[47] Guy W. (1976). ECDEU Assessment Manual for Psychopharmacology.

[48] Busner J., Targum S.D. (2007). The clinical global impressions scale: Applying a research tool in clinical practice. Psychiatry.

[49] Baldwin D.S., Chrones L., Florea I., Nielsen R., Nomikos G.G., Palo W., Reines E. (2016). The safety and tolerability of vortioxetine: Analysis of data from randomized placebo-controlled trials and open-label extension studies. J. Psychopharmacol..

[50] García-Alberca J.M., Gris E., Mendoza S. (2022). Effects of tianeptine treatment on depression and cognitive function in patients with Alzheimer’s disease: A 12-month retrospective observational study. J. Alzheimer’s Dis..

[51] Gonda X., Sharma S.R., Tarazi F.I. (2019). Vortioxetine: A novel antidepressant for the treatment of major depressive disorder. Expert. Opin. Drug. Discov..

[52] Dale E., Zhang H., Leiser S.C., Xiao Y., Lu D., Yang C.R., Plath N., Sanchez C. (2014). Vortioxetine disinhibits pyramidal cell function and enhances synaptic plasticity in the rat hippocampus. J. Psychopharmacol..

[53] Du Jardin K.G., Jensen J.B., Sanchez C., Pehrson A.L. (2014). Vortioxetine dose-dependently reverses 5-HT depletion-induced deficits in spatial working and object recognition memory: A potential role for 5-HT1A receptor agonism and 5-HT3 receptor antagonism. Eur. Neuropsychopharmacol..

[54] De Diego-Adeliño J., Crespo J.M., Mora F., Neyra A., Iborra P., Gutiérrez-Rojas L., Salonia S.F. (2022). Vortioxetine in major depressive disorder: From mechanisms of action to clinical studies. An updated review. Expert Opin. Drug Saf..

